# A Signal Processing Approach for Detection of Hemodynamic Instability before Decompensation

**DOI:** 10.1371/journal.pone.0148544

**Published:** 2016-02-12

**Authors:** Ashwin Belle, Sardar Ansari, Maxwell Spadafore, Victor A. Convertino, Kevin R. Ward, Harm Derksen, Kayvan Najarian

**Affiliations:** 1 Department of Emergency Medicine, University of Michigan, Ann Arbor, Michigan, United States of America; 2 Michigan Center for Integrative Research in Critical Care, University of Michigan, Ann Arbor, Michigan, United States of America; 3 Department of Mathematics, University of Michigan, Ann Arbor, Michigan, United States of America; 4 Department of Computational Medicine and Bio-informatics, University of Michigan, Ann Arbor, Michigan, United States of America; 5 College of Literature, Science, and the Arts, University of Michigan, Ann Arbor, Michigan, United States of America; 6 Combat Casualty Care Research Program US Army Institute of Surgical Research, San Antonio, Texas, United States of America; University of Washington, UNITED STATES

## Abstract

Advanced hemodynamic monitoring is a critical component of treatment in clinical situations where aggressive yet guided hemodynamic interventions are required in order to stabilize the patient and optimize outcomes. While there are many tools at a physician’s disposal to monitor patients in a hospital setting, the reality is that none of these tools allow hi-fidelity assessment or continuous monitoring towards early detection of hemodynamic instability. We present an advanced automated analytical system which would act as a continuous monitoring and early warning mechanism that can indicate pending decompensation before traditional metrics can identify any clinical abnormality. This system computes novel features or bio-markers from both heart rate variability (HRV) as well as the morphology of the electrocardiogram (ECG). To compare their effectiveness, these features are compared with the standard HRV based bio-markers which are commonly used for hemodynamic assessment. This study utilized a unique database containing ECG waveforms from healthy volunteer subjects who underwent simulated hypovolemia under controlled experimental settings. A support vector machine was utilized to develop a model which predicts the stability or instability of the subjects. Results showed that the proposed novel set of features outperforms the traditional HRV features in predicting hemodynamic instability.

## Introduction

Critical illness and injury is capable of leading to hemodynamic changes which, when left untreated, can lead to acute hemodynamic decompensation, shock, multi-organ failure and ultimately death. Advanced hemodynamic monitoring is a critical component of treatment in clinical situations where aggressive, yet guided interventions are required in order to stabilize the patient and optimize outcomes. Hemodynamic decompensation and compromise can occur for various reasons such as hypovolemia, sepsis, trauma, burns, heart failure, neurogenic injury, acute myocardial infarction, etc. While there are many tools at a physician’s disposal to monitor the patient in the hospital setting, the reality is that none of these tools allow hi-fidelity assessment of early physiologic changes that if recognized could result in earlier intervention and improvements in outcome. In fact, standard methods for assessing hemodynamic status, such as arterial blood pressure (BP), heart rate (HR) and arterial hemoglobin oxygen saturation (SaO2), can remain stable until the onset of overt conditions and thus often do not provide adequate actionable information that would enable physicians and care givers to perform the necessary early interventions that may avert pending decompensation [1, 2].

Critical illness and injury do not always result in an immediate change in vital signs that provide caretakers a warning that action must be taken immediately. The body is capable of producing impressive compensatory physiology which maintains vital signs in normal ranges until these compensatory mechanisms are exhausted, resulting in a state of decompensation. In hemorrhage, for example, depending on the injury and rate of blood loss, as much as 40% of circulatory blood volume could be lost before vital signs become overtly abnormal. [2] The ability to compensate, however, is specific to an individual and is based on many factors including basal state of health, aerobic capacity, etc [3].

Therefore, although there is a need for high-fidelity assessment of hemodynamic function that would allow for early intervention, most methods that rely on standard vital signs obtained from current technologies are severely limited [1] [4] [5].

Currently, there are research studies being conducted which attempt to determine hemodynamic stability using continuous non-invasive BP measurements and examination of arterial waveforms collected using devices such as Nexfin (formerly known as Modelflow, Finapress) [6, 7] pulse oximetry, and seismocardiogram [8]. While these approaches are showing promise, there is a need to exploit other readily obtainable signals such as the ECG.

As a result, there is a need for an improved set of features extracted from both HRV as well as the raw ECG signal which are designed to function specifically in quick and real-time monitoring settings with short windows of ECG signal, this study presents new options which could be useful, presenting certain novel features which are compared and contrasted against standard HRV based features. To test their effectiveness a database which contains physiological data of simulated hypovolemia in healthy volunteer subjects under controlled experimental settings was used.

The remaining sections are organized as follows: Section *Data* describes the physiological signal database used for this study. Section *Methods* contains multiple subsections describing the signal decomposition and feature extraction phase, including extraction of standard HRV based features and the new set of features presented in this study. Section *Results* describes the process of feature space reduction, machine learning modeling, and performance evaluation. Finally the *Discussion* section provides the conclusion from this study.

## Data

To develop, test and validate the proposed automated signal processing system for continuous monitoring of hemodynamic condition of patients, a specific database was utilized. This database consists of hi-fidelity physiological signals, which includes electrocardiogram (ECG) signal sampled at 500Hz, collected from volunteer subjects undergoing Lower Body Negative Pressure (LBNP), housed at the U.S. Army Institute of Surgical Research (USAISR) in San Antonio, TX. This model of LBNP has been established as a human model of compensated hemorrhage [3]. The data was collected by the USAISR under a research protocol and consent procedure that was sanctioned by its own internal review board (IRB). The participants provided a written consent to participate in this study. The USAISR was solely responsible for recording the consents and maintaining the records. Under a Cooperative Research and Development Agreement between the USAISR and the University of Michigan, a completely de-identified version of this dataset was provided by the USAISR for this study. This dataset consists of 200 subjects, all of whom were healthy volunteers recruited by the USAISR and none of whom had undergone any special conditioning prior to the study. These subjects were asked to refrain from exercise, alcohol and stimulants such as caffeine and other non-prescription drugs for the 24 hours prior to this experiment thereby mitigating any potential biases on their cardiovascular functions.

Central hypovolemia was simulated in the subjects by introducing increasing negative pressure to their lower body, performed using an LBNP chamber [9]. In this experiment, each subject is positioned such that the lower half of their body is inside the chamber while the upper half of the body is attached to multiple sensors that collect continuous physiological signals during the experiment. The LBNP protocol consisted of a 5-minute rest period (baseline) followed by 5 minutes of chamber decompression at 15, 30, 45, and 60 mmHg and then additional increments of 10 mmHg every 5 minutes until the onset of hemodynamic decompensation or the completion of 5 minutes at 100 mmHg. The attending investigator closely monitored each subject to determine the onset of hemodynamic decompensation, identified by a precipitous fall in systolic arterial BP concurrent with presyncopal symptoms such as bradycardia, gray-out (loss of color vision), tunnel vision, sweating, nausea or dizziness. The LBNP model used in this study provides a platform by which to examine the compensatory physiology of central hypovolemia and to design early warning systems that may allow caretakers advance warning prior to overt decompensation.

Among the 200 cases of subject data available, some were not utilized in this study. Some of the data collected had very low signal to noise ratio and some subjects were too intolerant of negative pressure, resulting in insufficient time in the chamber. Data from 178 subjects was utilized in the final analysis.

**Fig 1:**
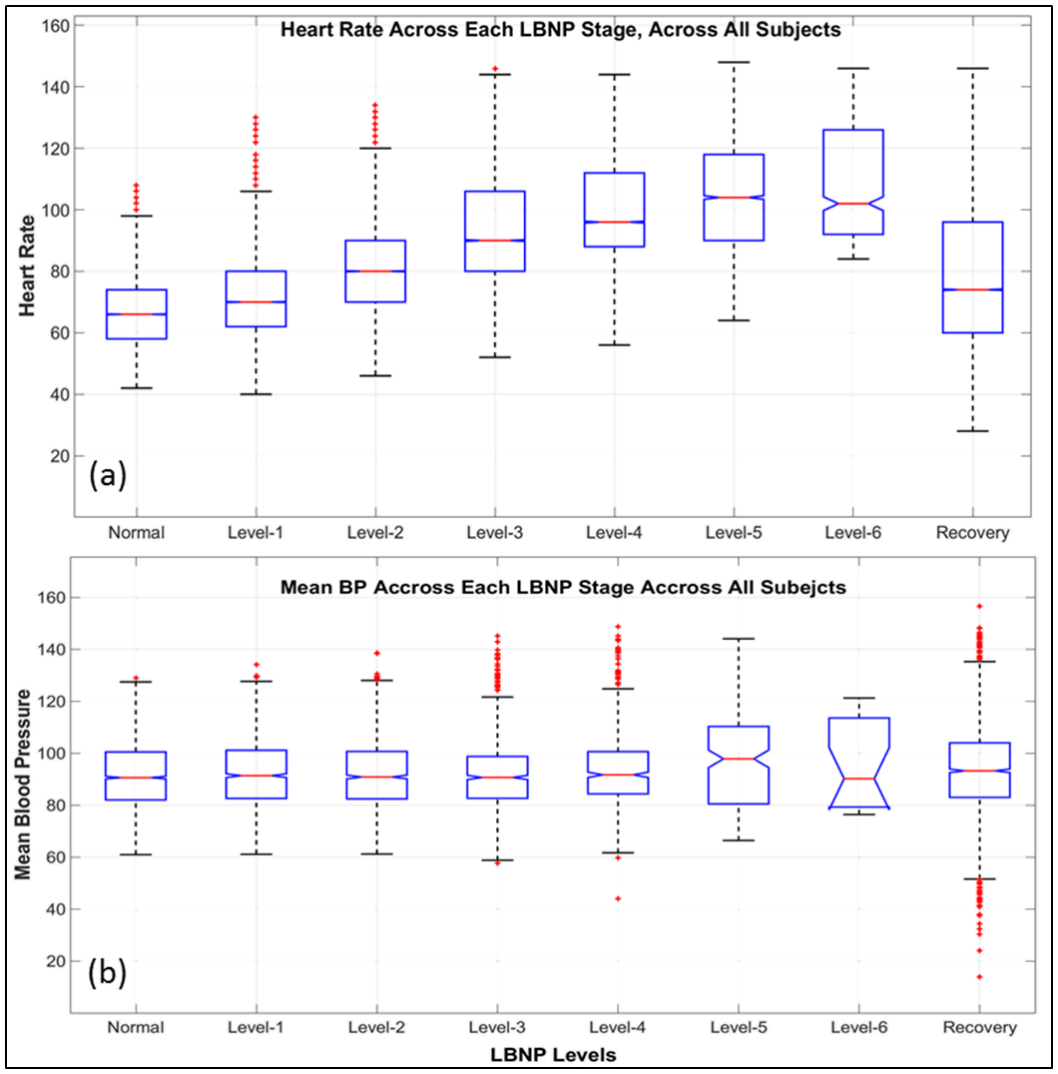
Heart Rate and Blood Pressure. The central mark within each box is the median, the edges of the box are the 25th and 75th percentiles, the whiskers are the standard deviations of the data at each stage and the outliers are plotted in red ‘+’. (a): Box plot depicting the interpersonal variability of HR in each stage across all subject; (b): Box plot of overall mean and standard deviation of arterial BP across each stage of the LBNP experiment.

During hemodynamic compensation to hypovolemia the information available with typical vitals such as BP and HR does not provide adequate actionable acumen for physicians and care givers to perform necessary interventions. Fig 1(a) and 1(b) show the variation of HR and mean BP across each stage for all the 178 subjects, respectively. For this study we have utilized the continuous waveform of the ECG lead-II only.

First, it can be seen that there is significant overlap in data at each level of the LBNP, both in HR as well as BP. The majority of the subjects remain within what is considered as a normal range of HR of 60–100 beats per minute (BPM) even during the later and more severe levels of LBNP. Similarly, the average mean BP of all the subjects remains within a range of 80–120 mmHg throughout the LBNP experiment. Second, there are large overlaps within the standard deviations of the data between subsequent stages of the LBNP. In this cohort, 56 individuals reached up to 4 levels of negative pressure (60 mmHg), 55 reached up to level 5 (70 mmHg), 47 reached level 6 (80 mmHg), 16 reached level 7 (80 mmHg) and 4 reached level 8 (100 mmHg). This suggests that there is a fairly large amount of inter-personal variability between these subjects. In fact, significant variation exists in the tolerance and compensatory reserve of individuals undergoing LBNP in this controlled model, with very few individuals capable of reaching 100 mmHg of LBNP. Due to these factors, using a simple rule based systems for recognizing severity of hemorrhage via traditional vital signs is not an efficient mechanism to detect onset of hemodynamic decompensation at an early stage. A further challenge to such an approach might be the need to know the baseline vital signs which is not always practical in real-life. It is evident that neither the BP nor the HR of these subjects changes overtly even during the severe stages of LBNP.

## Methods

**Fig 2:**
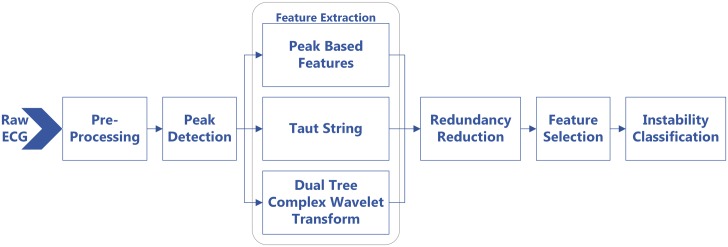
Methods Flowchart. Outline of the various discrete steps involved in the designed methodology.

Our methodology includes several steps executed in sequence as shown in Fig 2. Each of these steps are explained in detail in the following sections.

### Preprocessing

The first step in the preprocessing stage is *windowing*. Since the system is designed for application in real-time continuous monitoring settings, a tumbling window system has been utilized. After testing a variety of windowing methods, a beat size based windowing approach was then utilized where each window contains 120 complete beats of ECG. Other sizes of windows were also empirically tested and the results are shown in later sections. Each window of the ECG signal is modeled to fit a polynomial function of degree 6, where the optimal coefficients are determined by minimizing the sum of squared errors, and subtracted from the ECG signal to remove the baseline drift [10].

The next step in the preprocessing stage is *noise removal*. For this, the Savitzky-Golay filter has been utilized which is a generalized moving average filter with its coefficients determined by an unweighted linear least-squares regression and a second degree polynomial model [11]. The filtered signal is then used for peak detection.

### Peak Detection

After pre-processing, we detect the P, Q, R, S, and T peaks within the signal. To do this, the ECG window is first filtered using a level-10 wavelet transform and Daubechies 4 mother wavelet. This is done by setting the approximate coefficients and detail coefficients at level 10 to zero. This process maintains the shape of the signal and keeps the location and sharpness of the peaks intact while removing very low-frequency noise.

The filtered signal is then used to find the R peaks. The SQRS function in the PhysioNet toolbox, which is based on the algorithm in [12], has been used for this purpose. Next, the PhysioNet routine *ecgpuwave* was used to find the P, Q, S, and T peaks, and the R peaks that were found in the previous step were provided as inputs to the algorithm. Any missing P, Q, S and T peaks were augmented based on the timing of the same peak in the previous periods normalized by the ratio of the current and previous RR intervals. The results were visually inspected and the performance and accuracy of the detections were verified.

The ECG signals were normalized before feature extraction by subtracting the median of the signal and dividing by the mean of the R peak amplitudes. This allows us to later use the amplitude of the P, Q, S and T peaks as features.

By using a processing window for feature extraction that has a fixed length, some of the extracted features can become dependent on the HR. Our intention was to exclude HR from the feature set since including HR can lead to low specificity in practice (see Fig 1 and its description in Section *Data*). As a result, a variable window length was used for feature extraction that included a fixed number of beats rather than a fixed unit of time. As it is shown in Section *Model Performance*, a window that contains 120 beats is the best choice for the window size. The results that are presented in the rest of the paper are based on a window of 120 beats, unless stated otherwise.

### Feature Extraction

#### Peak-Based Features

The first set of peak-based features that were extracted are based on the normalized time intervals between the peaks. The *t*_*p*_(*i*), *t*_*q*_(*i*), *t*_*r*_(*i*), *t*_*s*_(*i*) and *t*_*t*_(*i*) are defined as the time of the P, Q, R, S and T peaks that are contained in the *i*^th^ sliding window, respectively. The feature *t*_*qt*/*rr*_ is defined as

$$\begin{array}{c} t_{qt/rr}=\frac{1}{n}\sum_{i=1}^{n}\frac{t_{t}\left(i\right)-t_{q}\left(i\right)}{t_{r}\left(i\right)-t_{r}\left(i-1\right)}, \end{array}$$ (1)

where *t*_*t*_(*i*) − *t*_*q*_(*i*) is the length of the *i*^th^ Q-T segment, and *t*_*r*_(*i*) − *t*_*r*_(*i* − 1) is the length of the *i*^th^ R-R interval. Likewise, all the possible combinations of the ratios of peak intervals were computed and included in the feature set. Note that all the time intervals are normalized by another interval to remove the effect of HR from the extracted features.

The second set of morphology-based features is computed using the amplitude of the peaks, denoted by *a*_*p*_(*i*), *a*_*q*_(*i*), *a*_*r*_(*i*), *a*_*s*_(*i*) and *a*_*t*_(*i*). The amplitude features *a*_*qt*_ and *a*_*rr*_ are computed as

$$\begin{array}{c} a_{qt}=\frac{1}{n}\sum_{i=1}^{n}a_{t}\left(i\right)-a_{q}\left(i\right), \end{array}$$ (2)

$$\begin{array}{c} a_{rr}=\frac{1}{n}\sum_{i=1}^{n}a_{r}\left(i\right)-a_{r}\left(i-1\right). \end{array}$$ (3)

Moreover, the ratio and interaction of the amplitude features are also included in the feature set.

$$\begin{array}{c} a_{qt/rr}=\frac{1}{n}\sum_{i=1}^{n}\frac{a_{t}\left(i\right)-a_{q}\left(i\right)}{a_{r}\left(i\right)-a_{r}\left(i-1\right)}, \end{array}$$ (4)

$$\begin{array}{c} a_{qt*rr}=\frac{1}{n}\sum_{i=1}^{n}\left(a_{t}\left(i\right)-a_{q}\left(i\right)\right)\left(a_{r}\left(i\right)-a_{r}\left(i-1\right)\right). \end{array}$$ (5)

Moreover, several non-typical features were extracted from the HRV signal. This includes features extracted from the Fourier transform and power spectral density (PSD) of HRV such as dominant frequency and its amplitude, median frequency and edge frequency. Finally, the respiratory signal was extracted from the modulations of the ECG signal, using the method suggested in [13] for single lead ECG, and several respiratory features were extracted. That includes the spread, dominant frequency and its amplitude, median frequency and edge frequency of the respiratory signal and its derivatives. A total of 839 features were extracted from the peak based computations alone.

#### Taut String

Once each of the QRS complexes have been located and the R peaks have been isolated, the HRV can be computed. If *r*_0_, *r*_1_, …, *r*_*n*_ are the times of the R-peaks as before, then the RR signal measures the lengths of the R-R intervals and is computed by

$$\begin{array}{c} z=\left(z_{1},z_{2},\cdots ,z_{n}\right)=\left(r_{1}-r_{0},r_{2}-r_{1},\cdots ,r_{n}-r_{n-1}\right)=D\left(r\right) \end{array}$$

where *D* is the difference operator and *r* = (*r*_0_, *r*_1_, …, *r*_*n*_).

For a fixed *ε* > 0, let *x* be the unique function such that

$$\begin{array}{ccc} {∥z-x∥}_{\infty } & = & \max_{i}\left\{\right|z_{i}-x_{i}\left|\right\}\leq \varepsilon \\ & & \text{and}\\ {∥D\left(x\right)∥}_{2} & = & \sqrt{\sum_{i=1}^{n-1}{\left(x_{i+1}-x_{i}\right)}^{2}} \end{array}$$

is minimal, and set *y* = *z* − *x*. The function *x* also minimizes

$$\begin{array}{c} {∥D^{⋆}D(x)∥}_{1}=|x_{2}-x_{1}|+\sum_{i=2}^{n-1}|x_{i-1}-2x_{i}+x_{i+1}\left|+\right|x_{n}-x_{n-1}|. \end{array}$$

where $D^{⋆}:R^{n-1}\to R^{n}$ is dual to $D:R^{n}\to R^{n-1}$ and is given by

$$\begin{array}{c} D^{*}\left(w_{1},w_{2},\cdots ,w_{n-1}\right)=(-w_{1},w_{1}-w_{2},w_{2}-w_{3},\cdots ,\\ \;w_{n-2}-w_{n-1},w_{n-1}) \end{array}$$ (6)

**Fig 3:**
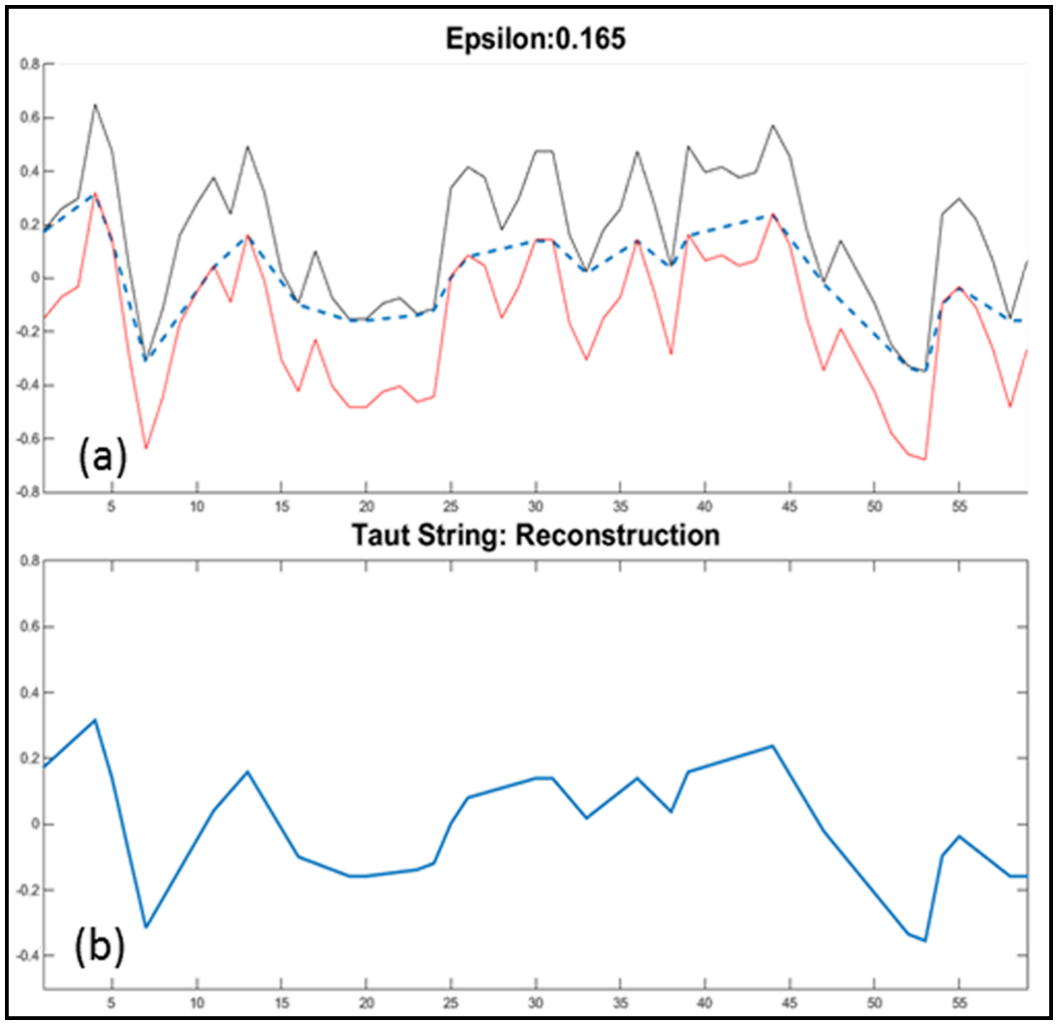
Taut String. X axis is the time in seconds (example shown is 60 seconds), Y axis is epsilon width from the origin for piecewise linear reconstruction. (a)The black and red waveforms are the taut-string margins, *z* − *ε* and *z* + *ε*, and the dotted blue lines is the taut-string estimation; (b) Shows the reconstructed signal based on the taut-string estimation.

We can think of *D*^⋆^ *D*(*x*) as a second derivative of *x* and ‖*D*^⋆^ *D*(*x*)‖_1_ as a measure of how much the function *x* bends. The function *x* can be computed using the Taut String Method, and we will write *x* = TS(*z*, *ε*) [14]. Graphically, the function *x* can be viewed as a string between *z* − *ε* and *z* + *ε* that is pulled tight (see Fig 3). The Taut String Method first appeared in statistics and was used to find a good ℓ_2_-approximation of a given function by a step function. An efficient algorithm for computing *x* = TS(*z*, *ε*) was described in [15] with a time complexity of *O*(*n*). Typically, *x* is a piecewise linear function that is smoother than *z*. We can consider the decomposition *z* = *x* + *y* as a denoising, where *x* is the denoised signal, *y* is the noise with ‖*y*‖_∞_ ≤ *ε* and *ε* is the noise level.

The variable epsilon *ε* is varied through a set range, and for each *ε* within the range we compute the decomposition *z* = *x*^(*ε*)^ + *y*^(*ε*)^, where *x*^(*ε*)^ = TS(*z*, *ε*).

Through the Taut String computation with different epsilon values, a total of 64 features were extracted. These are statistical measures such as mean, standard deviation, skewness, kurtosis, etc., computed on the Taut String estimation and the estimation error (the difference of the original signal and the estimation) at each epsilon as well as the inflection points of the Taut String estimates. After feature selection (Section *Feature Selection*), only features that were found to be independent and relevant to the development of the predictive model were retained. These features are explained in the Results section. (Section *Results*).

#### Dual Tree Complex Wavelet Decomposition

Analysis of certain time series signals, including ECG, in the time or frequency domain alone becomes insufficient for dynamic modeling. Transformations of such signals into the hybrid time-frequency domains can improve targeting and extracting of more salient features within the signal. A popular such transformation, which is often used to capture the signal characteristics in time and frequency domains, is the Short Time Fourier Transform (STFT) [16, 17]. However, STFT’s dependence on the shape and size of the window used in this transformation can greatly impact the quality and efficacy of signal representation [18]. Moreover, the use of sinusoidal basis functions, that span over the interval of [−∞,+∞] affects the ability and performance of STFT in extracting short-lived patterns in signals [18]. Also, the time-frequency precision of STFT is not optimal [19].

To combat this, another popular transformation that is often used is the Discrete Wavelet Transform (DWT) [20–22]. The wavelet transformation is based on a set of analyzing wavelets, each of which has its own time duration, time location and frequency band, allowing the decomposition of ECG signal into a set of coefficients [19]. The wavelet coefficient corresponds to a measurement of the ECG components in this time segment and frequency band. Despite DWT’s computational efficiency and sparse representation, the Wavelet transform suffers from a few fundamental and interrelated disadvantages such as complications during singularity extraction and modeling of signals, shift variance, aliasing, and lack of directionality [23–26]. So, applying DWT directly on the raw ECG waveform did not yield useful features.

To overcome some of these traditional shortcomings of DWT on ECG waveform, the Dual Tree Complex Wavelet Transform (DTCWT) technique has been employed for the signal decomposition and feature extraction [26, 27]. The DTCWT was first introduced by Kingsbury in 1998 [24]. In DTCWT, two non-complex DWT’s are used simultaneously at each decomposition level. The first DWT gives the real part of the transform while the second DWT gives the imaginary part. To perform the two streams of decomposition in the DTCWT framework, two complimentary filter banks are typically utilized. Detailed descriptions of these filters can be found in [28, 29]. The boundary issues, also known as shift variance which is typically found in standard wavelet methods, are mitigated to an extent with the DTCWT. The DTCWT is also approximately shift invariant, while offering both the magnitude and phase information of the signal.

Using DTCWT, each ECG window is decomposed to 5 levels with each level producing a real and imaginary component of the decomposed waveform. A final residual waveform is also obtained after all the waveform components are removed at each level form the original signal. In total, 198 features were extracted from each of the real and imaginary components of the 5 levels of decomposition. These features were mainly designed to capture the statistical as well as morphological variations of each level of the decomposition. After the feature selection steps (Section *Feature Selection*), only two features were found to be independent and informative for predictive model development, which have been described in the *Results* section.

#### Traditional HRV features

Several traditional HRV features which are commonly used by researchers and care-givers have also been extracted from the the signals for comparison [30–32]. These features have been derived in order to compare and contrast their effectiveness against the new set of features described in this study for the assessment of hemodynamic conditions of these subjects. These HRV features are extracted both from the time and frequency domains. From the time domain, the square root of variance of the R-R interval (a.k.a ‘NN interval’ in the medical community) (SDNN) is extracted which is defined as follows

$$\begin{array}{c} \text{SDNN}=\sqrt{\frac{1}{n-1}\sum_{j=1}^{n}{\left(z_{j}-\bar{z}\right)}^{2}}, \end{array}$$ (7)

where *z*_*i*_ is the value of the *i*-th R-R interval, and $\bar{z}=\frac{1}{n}\sum_{i=1}^{n}z_{i}$ is the average of the R-R intervals within the window. Since variance is mathematically equal to total power of spectral analysis, SDNN reflects all the cyclic components responsible for variability in the period of recording. The standard deviation of the first order differential (SDSD) of the R-R interval is computed as follows

$$\begin{array}{c} \text{SDSD}=\sqrt{\frac{1}{n-1}\sum_{j=1}^{n-1}{\left(|u_{i}|-\mu \right)}^{2}} \end{array}$$ (8)

where

$$\begin{array}{c} \left(u_{1},u_{2},\cdots ,u_{n-1}\right)=D\left(z\right)=\left(z_{2}-z_{1},z_{3}-z_{2},\cdots ,z_{n}-z_{n-1}\right) \end{array}$$

and $\mu =\frac{1}{n-1}\sum_{i=1}^{n-1}\left|u_{i}\right|$ is the average. The SDSD quantitatively captures the short-term variability within the HRV. Another measure similar to the SDSD is the root mean square of the successive differences (RMSSD) given by

$$\begin{array}{c} \text{RMSSD}=\sqrt{\frac{1}{n-1}\sum_{j=1}^{n-1}u_{i}^{2}} \end{array}$$ (9)

Another feature that is extracted is the pNN50, which is essentially the percentage of adjacent R-R intervals differing by 50 ms in the entire window. This is given by $\text{pNN}50=\frac{100}{n-1}\text{NN}50$, where

$$\begin{array}{c} \text{NN}50=\#\{i|1\leq i\leq n-1\;\text{and}\;|u_{i}|>50\text{ms}\}. \end{array}$$ (10)

is the number of times within the window that two consecutive R-R intervals differ by more than 50ms.

Some frequency-based features are also captured from the HRV. A power spectral density (PSD) is first computed for the R-R intervals using FFT based on the periodogram method [33]. The application is similar to the one used in the popular Kubios HRV analysis tool [34]. Using the computed PSD, powers of the low frequency (LF, 0.004–0.15 Hz) and high frequency (HF, 0.15–0.4 Hz) bands components are extracted. The HF/LF power ratio is also computed as an additional feature. Other extracted features include mean, kurtosis and the skewness of each HRV window.

## Feature Selection

Through the several feature extraction techniques mentioned thus far, there were 1117 different features that were computed and extracted from each window of the ECG signal. However, for the sake of brevity, only the features that have passed a feature selection process (described next) have been explained. Due to the extremely large number of features being extracted from each window, a feature selection process was required for eliminating those features that were redundant or not informative towards the machine learning model development.

Ideal feature sets for machine learning problems contain features that are highly correlated with the output but uncorrelated with each other. With a large, high-dimensional raw feature set (178 subjects, 1117 features per window of ECG), we applied a two-stage approach to feature selection. The first stage consisted of a redundancy reduction algorithm to remove multicollinearity from the large feature set, so that at the second stage, a stepwise forward selection method, could accurately reduce the feature set down to those described previously.

### Correlation Feature Redundancy Reduction

Multicollinearity, or high correlation between predictor variables, is problematic for linear regression analysis (and therefore our stepwise model selection method as well) because it causes regression coefficient estimates to become indeterminate [35]. To reduce feature-to-feature correlation, we employed an approach based on the correlation matrix. For each subject within the feature set, a correlation matrix *C*_*s*_ was calculated, where the *i*, *j*th element was the correlation between the *i*th and *j*th features of subject *s*, respectively. The mean correlation matrix *C*, which represented the average correlation between all features across all subjects was then calculated. This matrix was then thresholded, with an average correlation above the threshold being considered a *redundancy*. Redundancies for each feature were tallied, and redundant features were then removed from the feature set in greedy fashion, with the feature with the most redundancies removed first, the feature with the second highest removed second, and so on. Upon each feature removal, the redundancy counts for each feature were updated, as removal of a feature decreased redundancy counts for all the features it was correlated with. This continued until no features with redundancies at the given threshold level remained.

A feature-feature correlation of 0.9 or greater is commonly used as an indicator of multicollinearity [36]. We chose this value as our threshold, resulting in the removal of 392 features, for a remaining total of 725.

### Model Selection

The second and final step in reducing the size of the feature set was stepwise model selection. Choosing the best subset of the features with highest accuracy and lowest redundancy is a non-linear problem. Several methods have been proposed in the literature that use different criteria to find this subset [37, 38]. In this paper, sequential F-tests were used to select a small subset of the features which were highly correlated with the response variable but not with each other. This was done via a forward selection method with 10-fold subject-wise cross-validation. Starting with an empty model that only included an intercept, the feature that maximized the model R-squared on the training set was added in each step. The top 10 selected features were used for machine learning. The resulting model along with the selected features are described in the *Results* Section.

### Machine Learning

After feature selection, the selected features are then used to develop predictive models which can assess and predict the level of hemodynamic instability in the subjects, or in this case, the severity of the LBNP stage. For developing a predictive model, several different machine learning techniques, including discriminant analysis, nearest neighbors, decision trees, and support vector machine (SVM) were tested. Out of the ones tested, SVM consistently performed better than the rest and hence the results of SVM have been described in this section.

Each of the 178 subjects have an average of 12 windows of extracted features at window size of 120 beats. The feature set, when combined across all subjects, has a sum of 2085 rows across 10 columns. We found that the standard linear-kernel SVM, using the LIBSVM implementation, performed best [39]. We optimized the cost parameter *C* by performing 10-fold cross-validation for several different *C* values and then choosing the *C* which produced the model with the highest average area under the receiver operating curve (AUC) across all folds. If run on the entire dataset, however, this method risks overfitting the *C* parameter, because the testing set is used (and therefore exposed *before* model evaluation) when choosing *C*. To mitigate this, we perform an *outer* 10-fold cross-validation. A *validation* set is first taken from the dataset and set aside. The remaining data is then split into training and testing sets according to the cross-validation parameter optimization described above, with the best *C* for these training and test sets chosen. Then a new model is trained on the *combined* training and testing sets and evaluated on the validation set using the chosen *C*, producing metrics such as AUC, accuracy, sensitivity, specificity, etc. This process is repeated for each outer validation fold, and the metrics from each validation fold are averaged to produce the final metrics reported in this paper.

Since subjects decompensated at various LBNP stages, we created a 2-class description to provide more individual specific decompensation information. In this classification, we declared the beginning of early decompensation to occur half-way through each individual’s LBNP course. In the first half, individuals were considered adequately compensated. At the beginning of the second half, individuals were considered to be overtly decompensating. Identification of individuals at this point would still occur prior to any overt changes in vital signs, potentially allowing early identification and intervention [9]. Therefore the labels for machine learning were created using the levels of LBNP, where the overall levels of LBNP for each subject is divided into two equal parts. The first half of the LBNP stages including the baseline is labeled as class normal (subject is adequately compensated) and the second half of the data from the middle of the levels to the last but one level is considered as class abnormal (subject is becoming hemodynamically compromised). Such a strategy would still allow for recognition that the patient is becoming compromised prior to any overt changes in typical vital signs (HR and BP). In this study the recovery stage of the LBNP has been excluded. For the interest of a comparative study, the traditional HRV features and the new features described in this study are both used separately for developing independent models. The results are compared in the following section.

## Results

The selected features and their corresponding coefficient estimates and standard errors, t-ratios, p-values and variance inflation factors (VIF) are shown in Table 1. It is evident that all the selected terms are highly significant. Moreover, all the VIF values are below 10, indicating that the correlations between the terms are negligible.

**Table 1:** Table of selected features.

Index	Term	Estimate	Std Error	t-Ratio	Prob< |t|	VIF
	Intercept	0.9299624	0.073524	12.65	<.0001	.
F1	Kurtosis 3	-0.004307	0.000882	-4.88	<.0001	1.42
F2	Mean Coef 6	0.9385273	0.102607	9.15	<.0001	1.19
F3	Taut String 32	0.0112771	0.002347	4.8	<.0001	3.43
F4	*t*_*st*/*tt*_	-0.832286	0.213302	-3.9	0.0001	2.52
F5	*a*_pt*qq_	0.0003386	6.74E-05	5.03	<.0001	1.55
F6	*a*_pt*ss_	0.0002625	0.000072	3.64	0.0003	1.86
F7	*a*_rr*qq_	-0.000166	2.22E-05	-7.45	<.0001	1.16
F8	*a*_rt*tt_	0.0003911	0.000119	3.28	0.0011	1.70
F9	HRV Edge F.	0.0006793	0.000196	3.46	0.0006	2.63
F10	Resp Edge F.	-0.499431	0.062656	-7.97	<.0001	3.11

The 10 features that are selected by forward sequential cross-validated F-tests and their coefficient estimates, coefficient standard errors, t-ratios, p-values and variance inflation factors (VIF).

**Fig 4:**
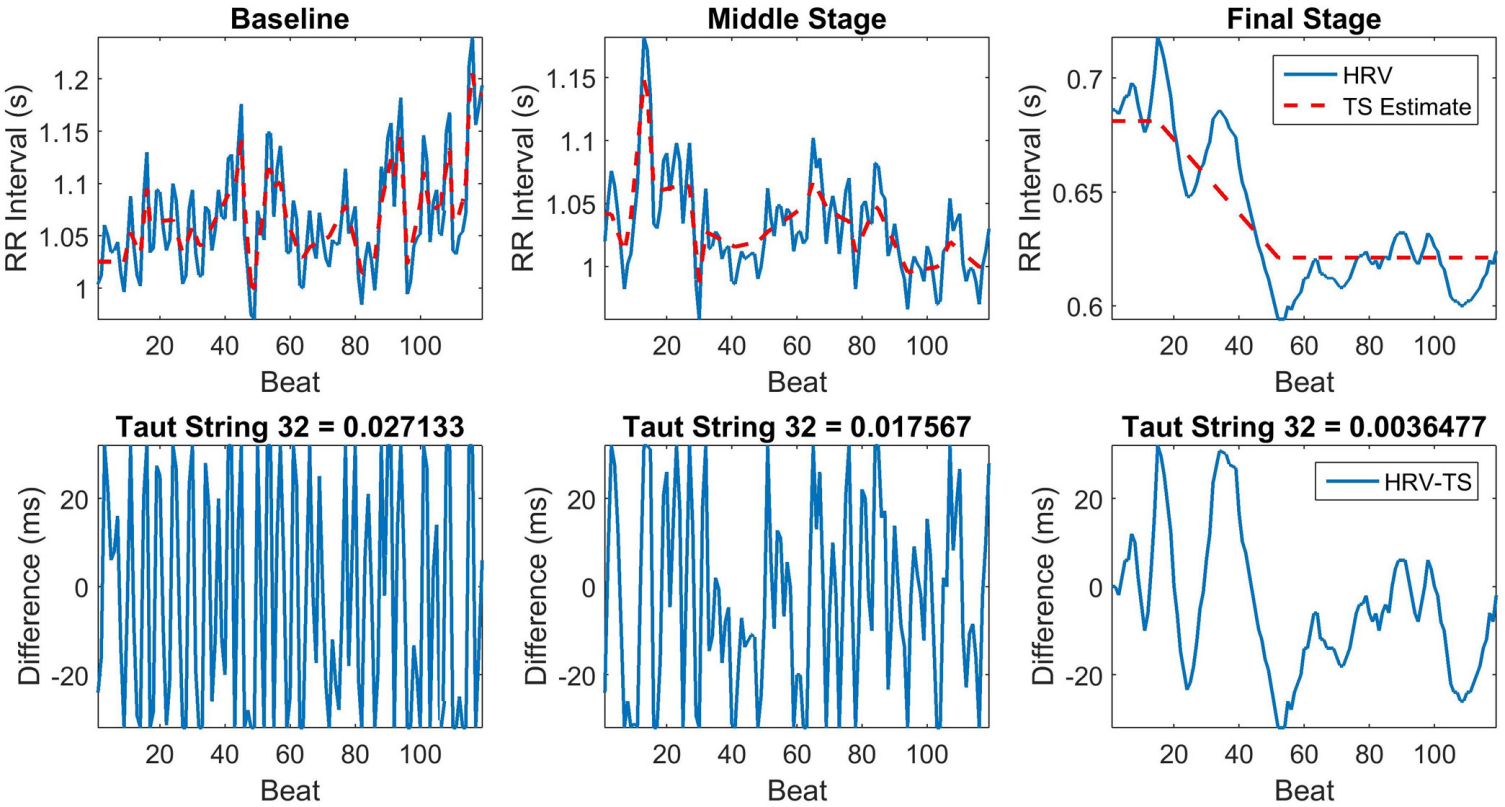
Top row. the HRV signals and their taut string estimates for a window at baseline, middle stage and final stage. **Bottom row**: the differences between the HRV waveforms and their taut string estimates. The figure also shows the value of the *Taut String 32* feature at each stage.

The first feature in Table 1, F1, is the Kurtosis of the residual signal from the 3rd level of DTCWT decomposition of the ECG signal. Kurtosis is essentially a measure of how outlier-prone a distribution is. The kurtosis of the normal distribution is 3. Distributions that are more outlier-prone than the normal distribution have kurtosis greater than 3; distributions that are less outlier-prone have kurtosis less than 3. The second feature F2 is the average value of the residual waveform obtained at the end of the 5 levels of DTCWT decomposition. Feature F3 in Table 1 is a Taut String feature which essentially is the average of the difference between the original HRV waveform and the taut string piecewise linear reconstruction of the waveform at an epsilon of 32ms. The HRV and its taut string estimate are shown in the top row of Fig 4 for the baseline, the middle LBNP stage and the final LBNP stage before the collapse. The differences between the HRV and the taut string estimate are shown in the bottom row. The subtraction removes the trend of the HRV waveform and only maintains the variations in the HRV that have an amplitude of or smaller than 32ms. The amount of variation seems to be correlated with the hemodynamic status and decreases as the subject approaches the point of collapse. The *Taut String 32* feature captures the level of variation by computing the mean absolute value of the derivative of the differences in the second row. The feature values are shown above each graph.

In addition, seven peak-based features were also selected. Feature F4 is the ratio of the S-T segment normalized by the T-T segment. Moreover, features F5–F8 are the amplitude interaction features that were selected. Finally, the edge frequency for the HRV and respiratory signals, F9 and F10, were added to the model. The edge frequency was computed based on the 95-th percentile of the PSD of the signals.

**Fig 5:**
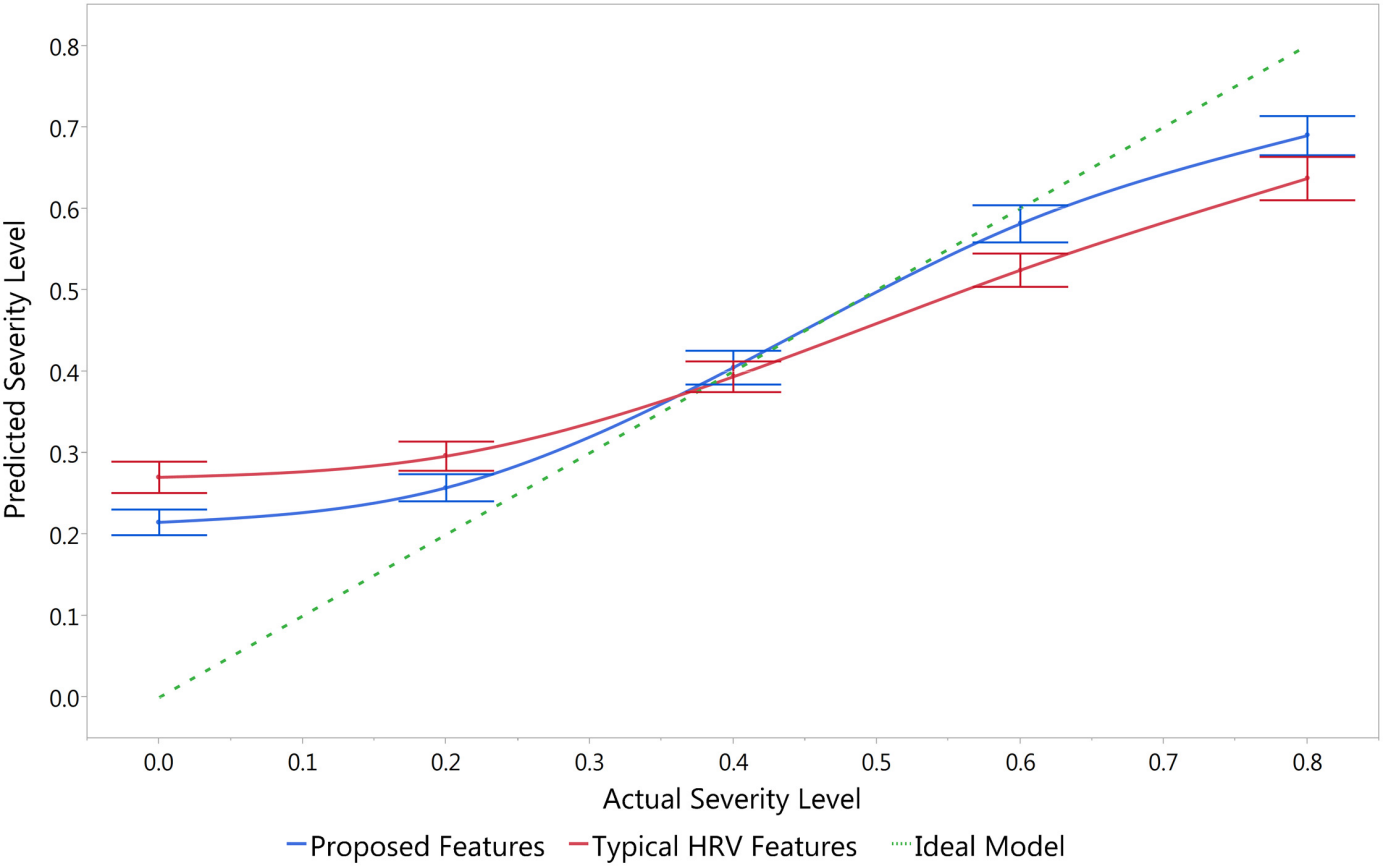
Predicted versus actual severity levels. The predicted severity levels are computed using multiple linear regression of the 10 selected features versus the typical HRV features. The bars indicate 95% confidence intervals.

A similar linear regression model was created using the 10 typical HRV features. The predicted severity levels are plotted against the actual severity levels for the two models in Fig 5. The severity level is defined as an index with a value of 0 at baseline and 1 at the point of collapse. Five severity levels are used from 0 to 0.8 (right before the collapse) and the LBNP stages are converted into these severity levels. The green horizontal line indicates an ideal model. The model with the proposed features (blue) is closer to the ideal line which indicates that the proposed features have a higher separation power compared to the typical HRV features (red). Moreover, the confidence interval bars indicate that the differences between the predicated values using the two models are statistically significant at all the levels except at 0.4. Notice that the model output at 0.4 is mainly determined by the intercept. Hence, the output values at 0.4 are expected to be equal while the largest separability occurs at the two extremes.

**Fig 6:**
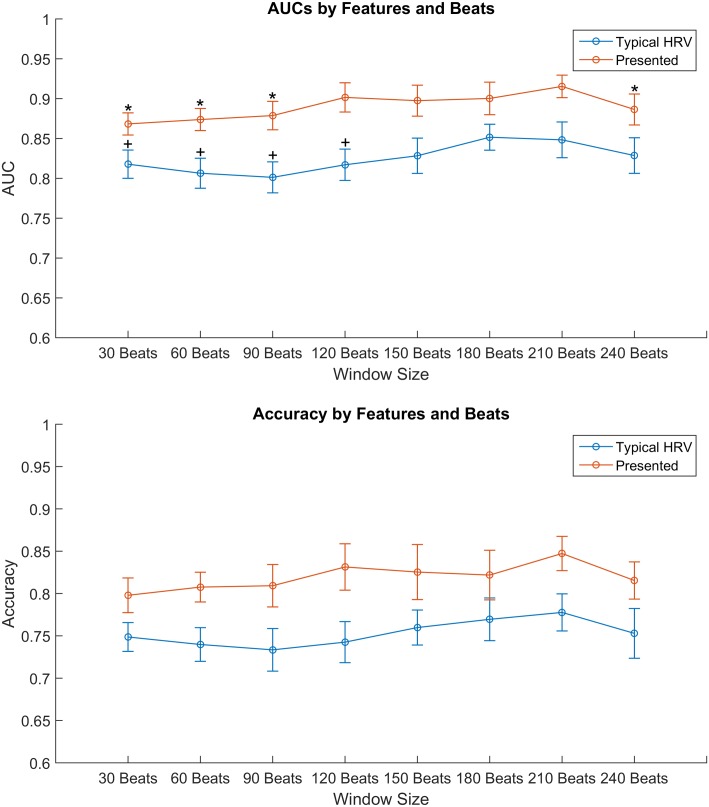
AUCs and Accuracies for Each Feature Set. (a) Areas under the receiver operating curve (AUCs). For the presented features, pair-wise Mann-Whitney U tests were used to compare the AUC’s for different window sizes to the one with the highest AUC (210-beats). The differences that are statistically significant are denoted by asterisks. The same was done for the typical HRV features and window sizes whose AUC was significantly different from the best window size (180-beats) are denoted by plus signs; (b) Accuracies averaged across all ten validation folds for each window size. Error bars represent a 95% confidence interval.

**Fig 7:**
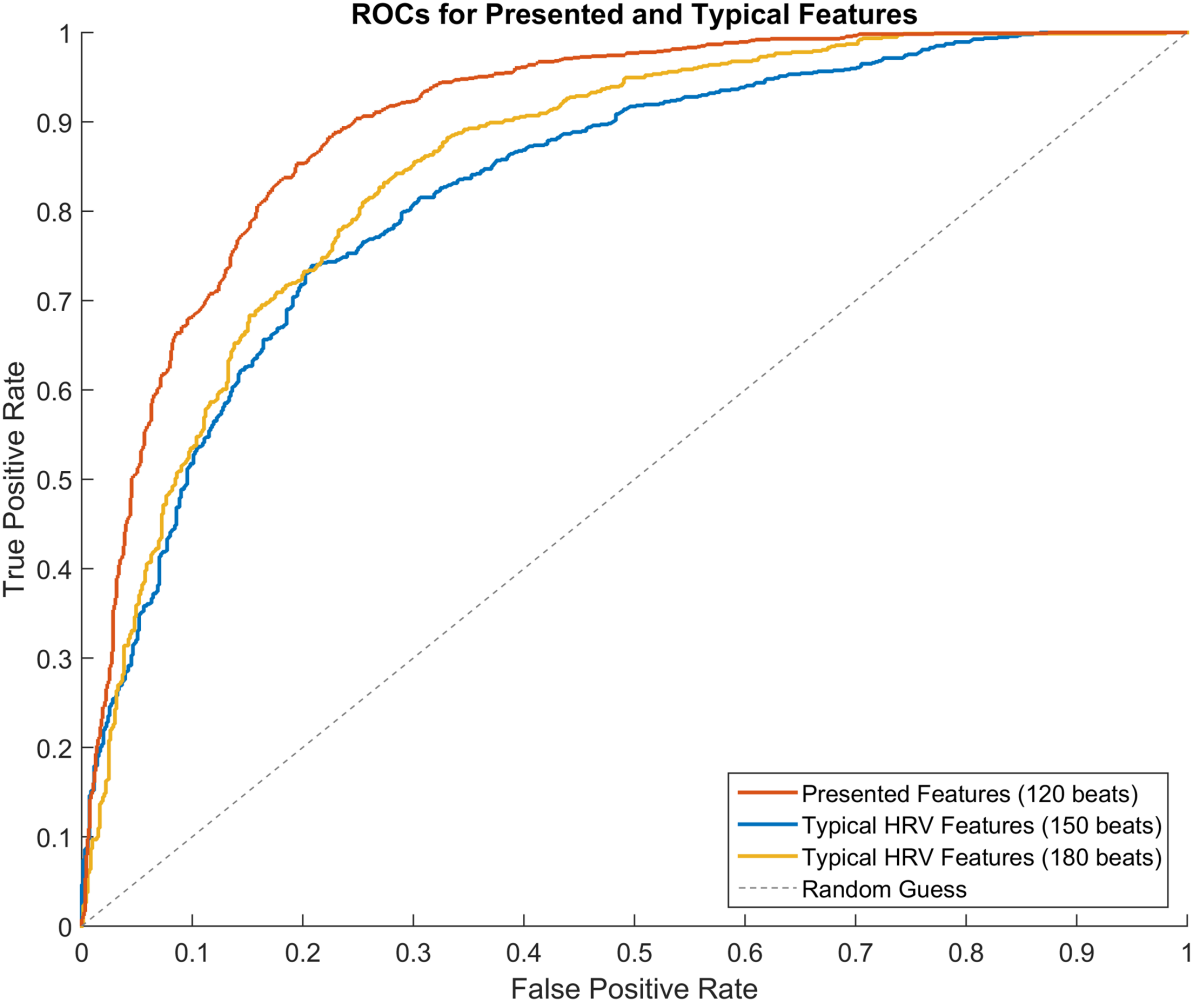
ROC. Receiver operating curves for each feature set’s highest-performing window size.

**Fig 8:**
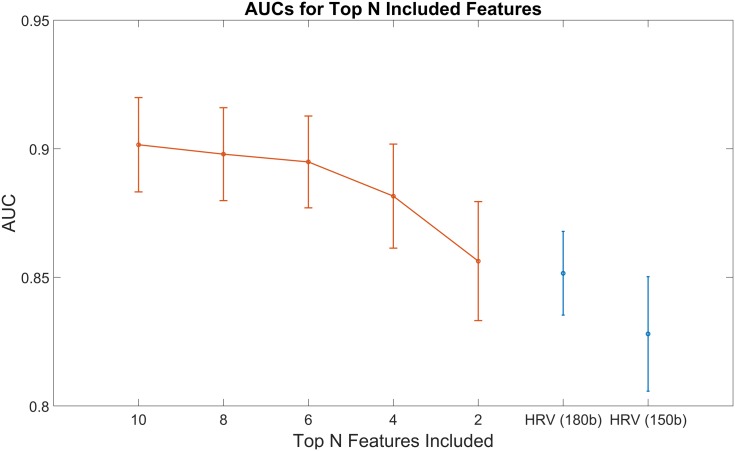
Featureset Sizes. Area under the curve calculated choosing the top ten, eight, six, four, and two features from the presented features’ best-performing window size (120-beats). The AUC for the Typical HRV features’ best performing window size (150-beats) is included (in blue) for comparison. The presented features continue to outperform the entire typical HRV feature set even when reduced to just four of the original ten features. The error bars indicate 95% confidence intervals.

To account for the fact that both the presented features and the typical HRV features may be better suited to capturing information from different lengths of ECG, we extracted both feature sets using window sizes from 30 to 240 beats with increments of 30 beats. The lowest baseline HR that exists in the dataset is 50 BPM, which prevents us from considering higher window sizes without losing a full stage. We then fit an SVM model for each specific window size and feature set combination according to the procedure outlined in the previous section. The resultant performance metrics are compared in Table 2 and Figs 6, 7 and 8.

**Table 2:** Prediction Model Comparison Table—The table compares the AUC, accuracy, sensitivity and specificity of the typical HRV features and the presented features for different window sizes as well as the 95% confidence intervals for the AUC and accuracy values. Asterisks indicate statistical significance at the *α* = 0.01 level. Bold indicates best score for that feature set. A non-parametric Mann-Whitney U test was used. The number of instances is denoted by n for each feature set.

Size	Features	AUC	Acc.	Sens.	Spec.
30 Beats	Typ. HRV	0.818 ± 0.018	74.866 ± 1.701	66.419	82.354
(n = 8689)	Presented	0.868 ± 0.014*	79.791 ± 2.048*	**74.983**	**84.051**
60 Beats	Typ. HRV	0.806 ± 0.019	73.978 ± 1.990	66.594	80.657
(n = 4233)	Presented	0.874 ± 0.014*	80.760 ± 1.760*	**74.640**	**86.305**
90 Beats	Typ. HRV	0.801 ± 0.019	73.347 ± 2.517	67.744	78.233
(n = 2779)	Presented	0.879 ± 0.018*	80.922 ± 2.507*	**75.080**	**86.081**
120 Beats	Typ. HRV	0.817 ± 0.020	74.258 ± 2.428	69.879	77.822
(n = 2085)	Presented	0.902 ± 0.018*	83.138 ± 2.741*	**77.642**	**87.696**
150 Beats	Typ. HRV	0.828 ± 0.022	75.982 ± 2.068	67.905	81.990
(n = 1655)	Presented	0.897 ± 0.019*	82.537 ± 3.255*	**76.770**	**86.717**
180 Beats	Typ. HRV	0.852 ± 0.016	76.953 ± 2.521	76.966	76.912
(n = 1506)	Presented	0.900 ± 0.020*	82.180 ± 2.932*	**78.076**	**86.028**
210 Beats	Typ. HRV	0.848 ± 0.022	77.769 ± 2.190	73.425	81.919
(n = 1321)	Presented	0.915 ± 0.014*	84.730 ± 2.024*	**81.937**	**87.468**
240 Beats	Typ. HRV	0.829 ± 0.022	75.293 ± 2.941	74.740	75.785
(n = 1156)	Presented	0.886 ± 0.019*	81.538 ± 2.198*	**78.759**	**84.074**

In Fig 6, the AUCs and accuracies are plotted for each feature set at each window size with their respective 95% confidence intervals. One can see that the presented features outperform the typical HRV features across all window sizes. Additionally, the presented features’ AUC and accuracy peak at the 210-beats window size. The asterisks indicate the window sizes whose mean AUCs are significantly different from that of the 210-beats window size according to pairwise Mann-Whitney U tests. Among the window sizes that are not significantly different from the maximum, the smallest window size, the 120-beats, is chosen as the best window size since it leads to the minimum diagnosis delay and has the least computational requirement. The AUC values at 120-beats and 210-beats windows are 0.902 and 0.915, respectively. On the other hand, the typical HRV features’ peak is at the 180-beats window size with an AUC of 0.852. The smallest window size with an average AUC that is not significantly different from the 180-beats is 150-beats with an average AUC of 0.828. The presented features outperform the typical HRV features with both 150 and 180-beats and all the differences were significant when tested using Mann-Whitney U tests.

In Table 2, the area under the receiver operating curve (AUC), accuracy, sensitivity, and specificity for each window size and feature set is provided. For the AUC and accuracy metrics, a non-parametric Mann-Whitney U-test was used to determine statistical significance for the presented features when compared to the respective typical HRV set with the same number of beats. The presented features outperformed the typical HRV features in terms of AUC and accuracy across all window sizes. Moreover, the selected model for the presented features (120-beats window) outperforms the best model for the typical HRV features (180-beats window) in terms of AUC and accuracy by 0.05 and 5.37%, respectively. Fig 7 shows the receiver operating curves (ROCs) for the presented features at 120-beats as well as the typical HRV features at 150- and 180-beats, with the presented features’ curve showing an increased true positive rate for every false positive rate.

In the interest of evaluating the performance of the models with fewer features, different models were developed with reduced featuresets based on feature ranking. In Fig 8, the AUCs calculated using the top-ranked ten, eight, six, four, and two features from the presented feature set at the 120-beats window size are plotted with 95% confidence intervals along with the AUC for the typical HRV features at 150- and 180-beats for reference. One can see that even a feature set consisting of only the top two of the presented features performs better than all the 10 typical HRV features, with the top four and above performing significantly better. Additionally, the figure demonstrates that the ten-feature set indeed performs best.

## Discussion

The presented features, coupled with the machine learning based severity categorization approach in this study, outperform the traditional HRV metrics in determining two classes of severity levels in subjects undergoing LBNP as a model of central hypovolemia consistent with hemorrhage. A significant aspect of the findings of this study is that in the final model, performance of the algorithm does not require knowledge of baseline patient data in order to accurately identify the subject as decompensating. The AUC and accuracy on our 120-beats window, for example, are based on using any 120-beats window regardless of what stage of LBNP the subject is experiencing. This is important since many situations resulting in sudden critical illness or injury, such as trauma, do not afford care providers an opportunity to trend vital signs or to know what a vicitm’s baseline vital signs are. Optimally, physiologic data obtained at any time point would be actionable. Developing such an automated continuous physiologic signal monitoring system may offer a unique and powerful method to monitor patients, thereby allowing the opportunity to identify the onset of hemodynamic instability at a time earlier than it would take to observe an overt and abnormal change in traditional vital signs.

This study has some obvious limitations. These include the fact that subjects were not actually injured or in pain during LBNP. Traumatic hypovolemia is, of course, accompanied by tissue damage and pain. It would be important to consider these factors and their effects on the ECG features extracted for this study. Whether the ECG features and machine learning algorithms developed for this data set will apply to other critical care states such as sepsis is not entirely clear but based on prevsious HRV science, our approach is likely to have implications.

In summary, this tool may offer advantages over traditional vital signs-based or current HRV-based monitors especially in cases where triage decisions need to be made within a very short period of time. The proposed system may enable high-fidelity analysis of ECG signals that are readily available under various echelons of care, from pre-hospital settings (i.e. ambulance transport), to the ICU, nursing home and even into the home environment where one of many popularly available portable ECG monitors can be made available.
